# The Book World of Medicine and Science

**Published:** 1898-10-08

**Authors:** 


### THE BOOK WORLD OF MEDICINE AND SCIENCE.

The Chemist's Compendium for Pharmacists, Medical Practitioners, and Students. By C. J. S. Thompson. Second edition. Revised and corrected by the British Pharmacopœia, 1898. (Whittaker and Co. London and New York. 1898. Pp. 313. Price 3s. 6d.)

The second edition of this most useful book has been brought out at a fitting time, following close upon the issue of the British Pharmacopœia of 1898. It is duly revised and brought up to date, and in addition to a complete synopsis of the formulæ and processes of the British Pharmacopœia, a table containing the strengths of the more powerful preparations of the British Pharmacopœia of 1898 is given. The book also contains a compact mass of useful tables, formulæ, methods, and analyses which render it one of the most generally useful of its kind, and much useful information on divers allied topics, *e.g.*, formulæ of English and foreign photographic chemicals, scales of medicines and medical appliances for merchant ships required by the Board of Trade, a list of modern remedies, and a table of doses for domestic animals. The book is exceedingly well got up, and cannot fail to be useful to all those for whom it is intended.

Cattle Tuberculosis. By T. M. Legge, M.D., and Harold Sessions, F.R.C.V.S. Pp. 77. Price 2s. 6d. net. (London: Baillière, Tindal, and Cox. 1898.)

Messrs. Legge and Sessions have here produced a work which, though intended in the first place for the farmer, butcher, and meat inspector, is of the greatest interest to all concerned with agriculture and public health. The names of the authors (Dr. Legge was Secretary to the Royal Commission on Tuberculosis in 1886-90) are alone sufficient guarantee of the accuracy of the facts and the sanity of the views put forward. While fully recognising the desirability —nay, the necessity—of some check on the sale of tuberculous carcases as food, Messrs. Legge and Sessions believe that, as a source of human infection, the flesh is less harmful than the milk of tuberculous animals. Consequently they recommend the close supervision by sanitary authorities of all dairies and cowsheds and the elimination of tuberculous stock by the use, for diagnostic purposes, of tuberculin supplied from official sources. That these measures may be made less distasteful to the farmer they recommend schemes of mutual insurance, and the sale, under certain restrictions, of the flesh of only partially diseased animals. A clear and excellent account is given, in language that will carry conviction and information to the farmer, of the nature, prophylaxis, and dangers of cattle tuberculosis. It is not without a certain sense of humiliation that we read in these pages how Denmark, Belgium, Sweden, and Norway—all poor countries—have provided adequate and elaborate machinery for the preservation of the public health and the gradual elimination of cattle tuberculosis, while Great Britain yet hesitates to even appoint skilled meat inspectors. In many Continental countries, too, as is now well known, tuberculin is supplied by the Government authorities, and in some its use is enforced by statute. Abroad, too, it is recognised that the inspection of meat can only properly be carried out by skilled veterinarians, and that legislative precautions must be taken to prevent the dissemination of tuberculosis by milk. In Germany arrangements have been made whereby carcases of animals only locally infected are cut up and sterilised by steam. The meat is then allowed to be sold at reduced rate on a special stall, known as a freibank. By this plan the very poor, who otherwise would be unable to obtain meat at all, can obtain it in a not unwholesome condition. At the same time, some satisfaction is afforded to the butchers, who would suffer severe loss were meat, purchased in good faith, to be confiscated without compensation. So popular has the "freibank" become that in Prussia one is established at at least five-sixths of the public abattoirs. It is hardly likely that in Great Britain popular clamour will ever demand legislative interference in these matters to the extent that is practised on the continent. But if scientific opinion is backed by a popular sense of the necessity for some improvement on the present state of things, much may be done. And if, as we trust, Messrs. Legge and Sessions' most valuable book attains a wide circulation amongst the classes to which it is addressed, we have no doubt that it will bring forth much fruit in due season.

Tests and Studies of the Ocular Muscles. By E. E. Maddox, M.D., F.R.C.S.E. Pp. 418. Illustrated. (Bristol: J. Wright and Co. 1898. Price 10s. 6d. net.)

This book is one of considerable importance, and is devoted to the elucidation of some of the most intricate and complex problems in ophthalmic science, namely, those concerning normal and abnormal ocular movements. The subject matter proper is prefaced by an excellent anatomical account, drawn chiefly from Motais' papers, in which not a few points are made that will be unfamiliar to most English readers. We then pass to an explanatory discussion of Donders and Listing's laws, and a very full account of the difficult subject of false torsion, in which the author, with much ability, reconciles the views of Helmholtz and Donders. The author also describes his very ingeniously-devised apparatus for the estimation of torsion angles. Individual ocular muscles, their lines of force, muscular planes, and axes of rotation are discussed, and the uses of Landolt's ball explained. The various forms of associated muscular action are elaborately detailed, and the ground cleared for a discussion of "Squints and Paralysis." The author dwells with fondness on the use of the tangent strabismometer, but other matters are fairly treated. The advantages of observing ophthalmoscopic corneal images are fully insisted on, and the book concluded by chapters on suppressed squint, latent torsion, and the eye in darkness. All these subjects are discussed in such detail, from both the practical and theoretical points of view, that probably few but specialists will care to study Dr. Maddox's book. Yet the style is so easy, and the author's meaning so readily grasped, that the complexity of the problems presented is forgotten in the charm and lucidity of the demonstrations. Much careful research and originality of thought can be traced in almost every chapter, and the diagrams often display extreme ingenuity. The only quarrel we have with the author is that his phraseology is sometimes hardly precise enough for discussions involving so much physical and mathematical detail. It is not, for instance, desirable to speak of "the two senses in which a body can rotate about one axis," when by "sense" direction is meant. But Dr. Maddox's book is singularly free from the inaccuracies and slips which so often detract from the value of books of this nature, and the author deserves the highest commendation for the way in which he has made pleasant what is too frequently left obscure and bewildering.

